# Shed syndecan-2 enhances tumorigenic activities of colon cancer cells

**DOI:** 10.18632/oncotarget.2885

**Published:** 2015-02-17

**Authors:** Sojoong Choi, Youngsil Choi, Eunsung Jun, In-San Kim, Seong-Eun Kim, Sung-Ae Jung, Eok-Soo Oh

**Affiliations:** ^1^ Department of Life Sciences and the Research Center for Cellular Homeostasis, Ewha Womans University, Seoul 120–750, Republic of Korea; ^2^ Center for Theragnosis, Biomedical Research Institute, Korea Institute of Science and Technology, Seongbuk-gu, Seoul 136–791, Korea; ^3^ Department of Biochemistry and Cell Biology, School of Medicine and Cell & Matrix Research Institute, Kyungpook National University, Daegu 700–422, Republic of Korea; ^4^ Department of Internal Medicine, Ewha Womans University School of Medicine, Seoul 158–710, Republic of Korea

**Keywords:** Colon cancer, shedding, signal transduction, syndecan-2, tumorigenesis

## Abstract

Because earlier studies showed the cell surface heparan sulfate proteoglycan, syndecan-2, sheds from colon cancer cells in culture, the functional roles of shed syndecan-2 were assessed. A non-cleavable mutant of syndecan-2 in which the Asn^148^-Leu^149^ residues were replaced with Asn^148^-Ile^149^, had decreased shedding, less cancer-associated activities of syndecan-2 *in vitro*, and less syndecan-2-mediated metastasis of mouse melanoma cells *in vivo*, suggesting the importance of shedding on syndecan-2-mediated pro-tumorigenic functions. Indeed, shed syndecan-2 from cancer-conditioned media and recombinant shed syndecan-2 enhanced cancer-associated activities, and depletion of shed syndecan-2 abolished these effects. Similarly, shed syndecan-2 was detected from sera of patients from advanced carcinoma (625.9 ng/ml) and promoted cancer-associated activities. Furthermore, a series of syndecan-2 deletion mutants showed that the tumorigenic activity of shed syndecan-2 resided in the C-terminus of the extracellular domain and a shed syndecan-2 synthetic peptide (16 residues) was sufficient to establish subcutaneous primary growth of HT29 colon cancer cells, pulmonary metastases (B16F10 cells), and primary intrasplenic tumor growth and liver metastases (4T1 cells). Taken together, these results demonstrate that shed syndecan-2 directly enhances colon cancer progression and may be a promising therapeutic target for controlling colon cancer development.

## INTRODUCTION

Colorectal cancer, an epithelial cancer which is localized to the colon or the rectum, is a significant issue in modern society. Over the last decade, great progress has been made in our understanding of the molecular mechanisms underlying colorectal cancer development. Associated mutations in various oncogenes, tumor suppressor genes, and mismatch repair genes have been identified [1, 2] and interactions among these genes have been shown to lead to uncontrolled neoplastic cell division and metastasis [3, 4]. In addition, efforts have been made to develop methods for colon cancer diagnosis and therapeutics. Classical markers for blood tests of malignancy, including prostate-specific antigen, carcinoembryonic antigen, CA19–9, and α-fetoprotein have been widely used as diagnostic indicators of colon cancer in spite of their significant shortcomings [5]. However, none of the methods are proven to diagnose colon cancer effectively. Therefore, discovery of colorectal cancer markers for diagnosis and therapeutics would be significant in improving a patient's survival.

Syndecan-2, a cell surface heparan sulfate proteoglycan, is mainly expressed on mesenchymal cells and plays a role in several cell regulation processes through cell-extracellular matrix adhesion [6]. One of the best examples of syndecan-2 functions is its regulatory role in cancer progression. High syndecan-2 expression is related to tumorigenic behaviors through regulation of cell adhesion, proliferation, and migration in colon cancer cells. Syndecan-2 interacts with various ligands and extracellular matrix proteins as docking receptors [6]. For example, syndecan-2 can regulate the localization and activation of matrix metalloproteinase (MMP)-7 [7], which is overexpressed in colon cancer [8]. Moreover, activated MMP-7 can enhance syndecan-2 extracellular shedding to produce the soluble form of syndecan-2 in colon cancer cell conditioned media [9]. Furthermore, MMP7 is known to regulate homotypic adhesion of colon cancer cells and enhance their metastatic potential [10].

Recent studies have demonstrated that syndecans can be shed in various cancers, including lung, breast, colon cancer and myeloma [11–16]. This cell surface receptor is proteolytically released from the cell surface as a soluble ligand-like growth factor, which gives it another function. Studies have shown that the shed syndecan extracellular domain is detected in body fluids and that shed syndecan levels are associated with tumor progression [14]. For example, the expression and shedding of syndecan-1 is upregulated in tumor cells, with the shed portion comprising an intact extracellular domain bearing the extracellular portion of the core protein and heparan sulfate moieties [17]. Shed syndecan-1 can be incorporated into the bone marrow extracellular matrix, which supports myeloma [18] or may remain as a soluble component within the bone marrow plasma [19]. Most importantly, high serum levels of syndecan-1 reflect a high tumor burden and predict poor prognosis in myeloma patients [19, 20]. Proangiogenic factors were found to stimulate the shedding of syndecan-2 during glioma tumorigenesis, and shed syndecan-2 was found to promote angiogenic processes [21]. In addition, the syndecan-4 extracellular domain was shed into acute human dermal wound fluids [22]. These findings collectively suggest that syndecan shedding can occur in cancer cells, and may impact cancer progression. In this study, we determined if shed syndecan-2 played a role in cancer regulation and if it could be used as a therapeutic biomarker for colon cancer.

## RESULTS

### Extracellular shedding is necessary for syndecan-2 mediated pro-tumorigenic functions

**Figure 1:**
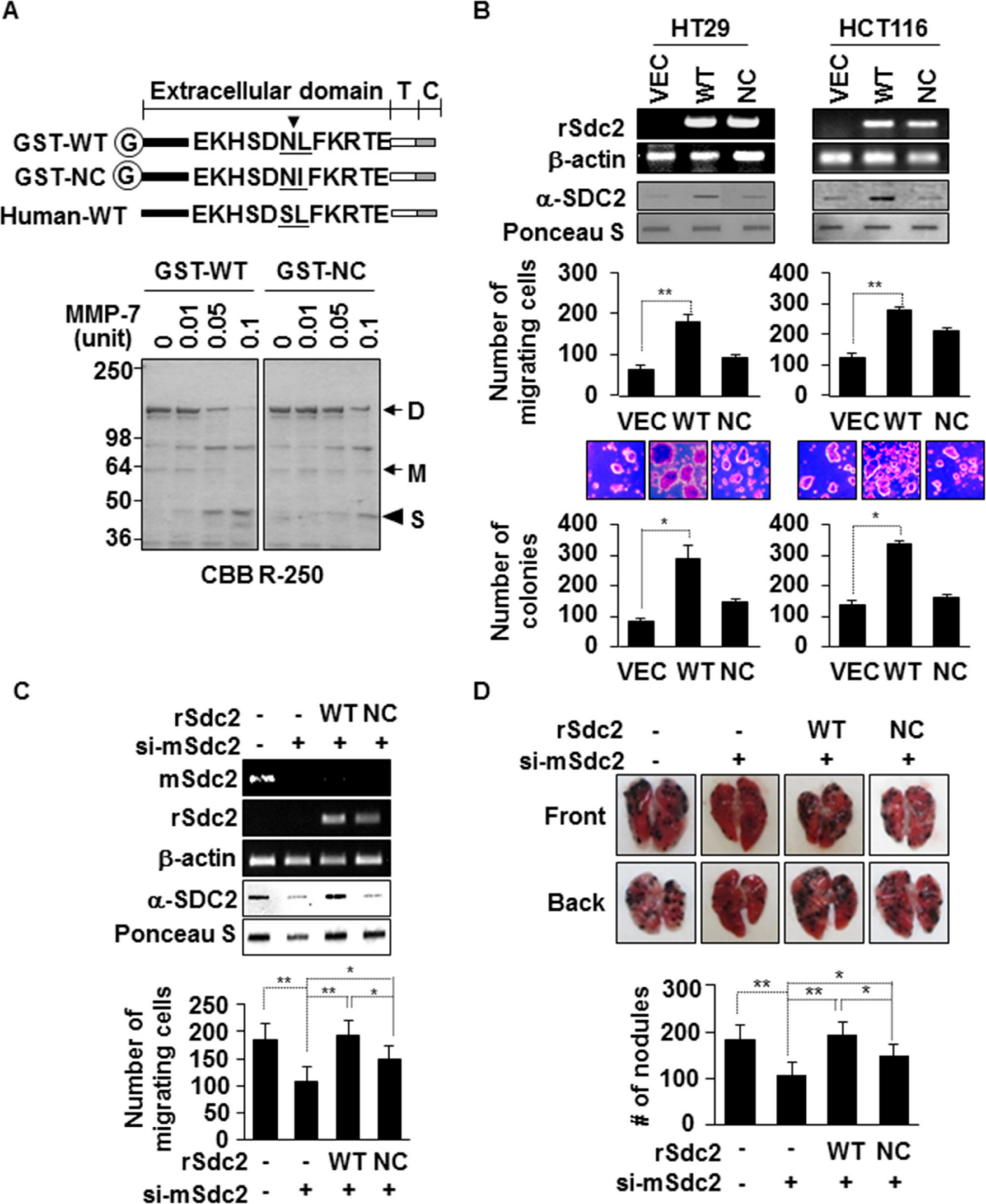
Extracellular domain shedding is necessary for syndecan-2 functions in colon cancer **(A)** Equivalent amounts of GST-fused rat syndecan-2 wild-type (WT) or the Leu^149^® Ile non-cleavable mutant (NC) were incubated with pro-MMP-7, separated by SDS-PAGE, and stained with Coomassie Brilliant Blue. The arrows indicate the dimeric (D) and monomeric (M) forms of syndecan-2. The arrowhead indicates the shed fragment of syndecan-2 (S). **(B)** Colon cancer cells were transfected with 1 μg of VEC, WT- or NC-syndecan-2. Syndecan-2 mRNA expression was evaluated by RT-PCR using the indicated primer. Conditioned medium was subjected to slot blotting with the anti-syndecan-2 antibody (top). Transfected cells were allowed to migrate on gelatin-coated (10 μg/ml) Transwell plates, and migrated cells were stained with hematoxylin and eosin, and counted (middle). *n* = 3; **p < 0.01. Transfected cells were added to bottom agar and colonies were counted after 2 weeks (bottom). *n* = 5; *p < 0.05. **(C)** B16F10 cells were transfected with siRNA targeting mouse syndecan-2 alone or with 1 μg of the indicated rat syndecan-2 constructs. Syndecan-2 mRNA expression, shed syndecan-2 in the conditioned media and cell migration were analyzed as described in Figure 1B. *n* = 5; **p < 0.01, *p < 0.05. **(D)** BALB/c mice were injected with the indicated B16F10 melanoma cells. After 2 weeks, lungs were removed and examined. Two independent experiments were performed (*n* = 5/each group). Representative photographs of the front and back sides of each lung are shown (top). The bar graph indicates the numbers of metastatic lung nodules (bottom). The columns represent the mean (± s.d.) number of lung metastatic nodules (*n* = 3). *p < 0.05, **p < 0.01.

To determine whether extracellular shedding of syndecan-2 was necessary for syndecan-2-mediated functions in colon cancer, we constructed a non-cleavable mutant (NC) of rat syndecan-2 in which the Asn^148^-Leu^149^ residues were replaced with Asn^148^-Ile^149^. Recombinant syndecan-2 was directly cleaved N-terminally at Leu^149^ of the extracellular domain by MMP-7. While 0.05 units of MMP-7 could cleave 50% of recombinant wild-type syndecan-2, non-cleavable-syndecan-2 reduced this cleavage by 5-fold (Figure 1A). As expected, the levels of shed syndecan-2 increased in HT29 and HCT116 cells transfected with a vector expressing WT-SDC2, compared with vector-transfected cells, but levels were much less in cells expressing a NC-SDC2 (Figure 1B). Cell surface expression of syndecan-2 was higher in transfected cells expressing the non-cleavable mutant compared to wild type syndecan-2 (Supplementary Figure S1), suggesting the reduced shedding of the non-cleavable mutant. Although cell migration was markedly increased in HT29 and HCT116 cells transfected with a vector expressing WT-SDC2, this syndecan-2 mediated cell migration was dramatically reduced in cells expressing NC-SDC2 (Figure 1B). Syndecan-2-mediated anchorage-independent growth was also significantly reduced in cells expressing NC-SDC2 (Figure 1B). Similarly, we could detect shed syndecan-2 in the conditioned media from B16F10 cells which are known to express MMP-7 (Supplementary Figure S2A [24]). The B16F10 cells overexpressing syndecan-2 showed increased cell migration, while those expressing mutant syndecan-2 showed reduced ability to migrate (Supplementary Figure S2B and Figure 1C). To analyze the role of the syndecan-2 extracellular domain shedding *in vivo*, mouse syndecan-2 (mSdc2) in B16F10 cells was knocked down using mouse specific siRNA [25] and then re-expressed using vectors encoding the rat syndecan-2 (rSdc2) cDNA. These B16F10 cells were injected into 6-week-old BALB/c mice via the tail vein to establish an experimental lung metastasis model. Analysis of lung samples revealed that mice receiving NC-SDC2-transfected B16F10 cells developed significantly fewer pulmonary metastases compared to those receiving WT-SDC2-transfected cells (Figure 1D). These data indicate that syndecan-2-mediated tumorigenic activity is related to its shedding.

### Shed syndecan-2 extracellular domain contributes to syndecan-2-associated cancer activity regulation

**Figure 2:**
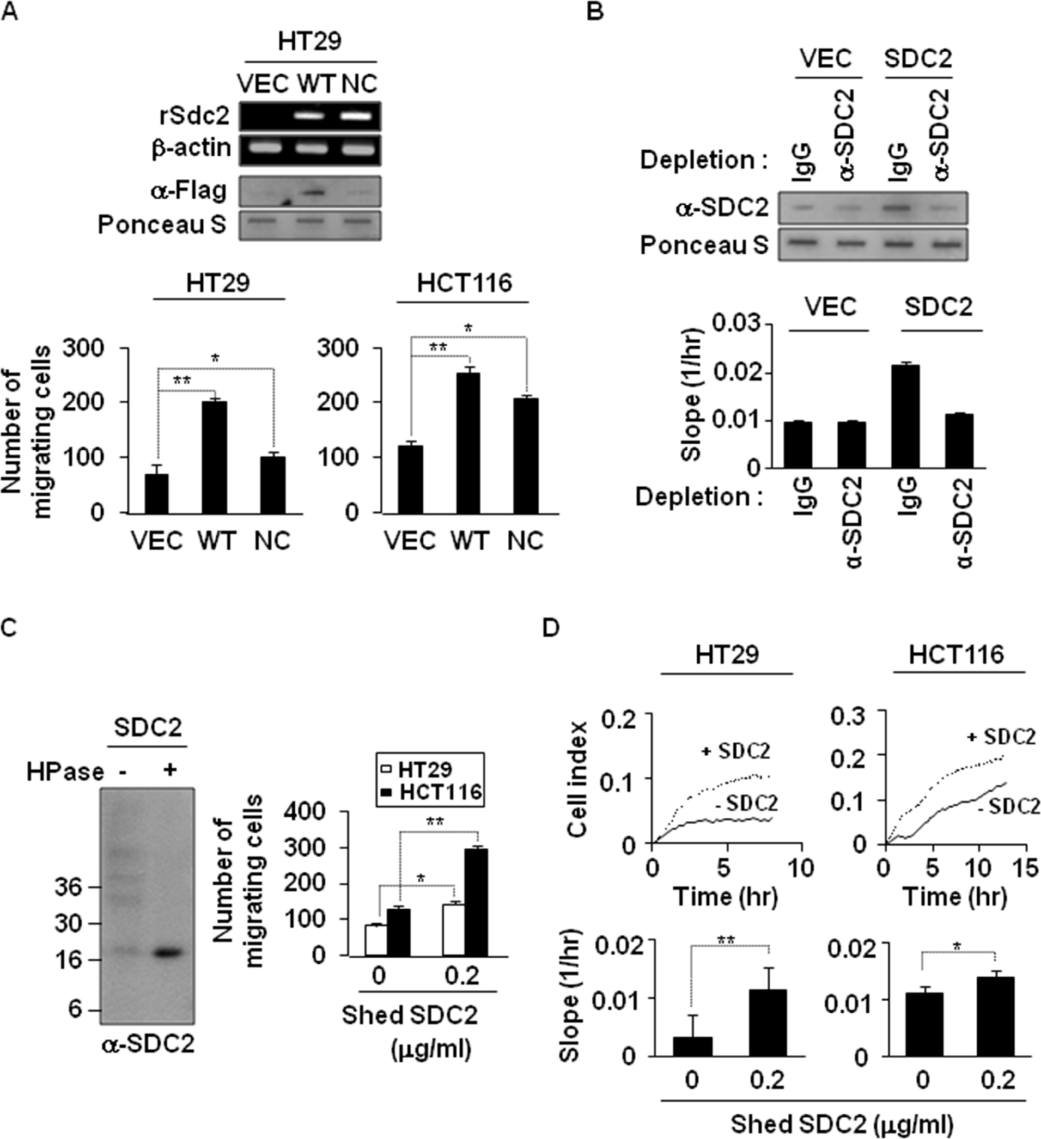
Shedding of syndecan-2 plays a critical role in colon cancer cell migration **(A)** HT29 cells were transfected with indicated cDNAs, and syndecan-2 mRNA expression was evaluated by RT-PCR. Conditioned media were subjected to slot blotting with the anti-Flag antibody (top). HT29 and HCT116 cells were treated with HT29 conditioned media (final 10% v/v) from VEC, WT-or NC-syndecan-2 mutant transfected cells and allowed to migrate on Transwell apparatus (bottom). *n* = 5; *p < 0.05, **p < 0.01. **(B)** Conditioned media were immunodepleted with control IgG- or anti-syndecan-2 antibody-conjugated protein G beads. The supernatants were subjected to slot blotting with anti-syndecan-2 antibody (top). Mixture of HCT116 cells with each supernatant (final 10% v/v) were added to the upper chambers of CIM-plates and migration curves were monitored using the xCELLigence system. The rates of cell migration over 24 hr were analyzed using the RTCA software to each RTCA CIM-Plate wells (bottom). **(C)** Shed syndecan-2 in HT29 cell conditioned media was isolated by DEAE-Sepharose column chromatography. Final elution fractions were digested by heparinase and analyzed by immunoblotting using anti-syndecan-2 antibody (left). Cells were treated with 0.2 μg/ml of purified shed syndecan-2, and Transwell migration assay was performed (right). *n* = 5; *p = 0.05, **p < 0.01. **(D)** Cells were treated with 0.2 μg/ml of purified shed syndecan-2, and a real-time migration assay was analyzed by xCELLigence system. *n* = 5; *p = 0.05, **p < 0.01.

To directly assess the role of syndecan-2 extracellular domain shedding, we investigated whether shed syndecan-2 itself promotes colon cancer cell activities. The level of shed syndecan-2 was increased in HT29 cells transfected with a vector expressing Flag-tagged WT-SDC2, compared with vector-transfected cells, but not in cells expressing Flag-tagged NC-SDC2 (Figure 2A, top). Cell migration was markedly increased in HT29 and HCT116 cells treated with WT-SDC2-expressing HT29 cell conditioned media (Figure 2A, bottom). Consistently, treatment with WT-SDC2-expressing HT29 cell conditioned media enhanced the migration of HCT116 cells, but depletion of shed syndecan-2 in the conditioned media abolished increased cell migration of HCT116 cells (Figure 2B). In addition, purified shed syndecan-2 from HT29 cell conditioned media (Figure 2C, left) directly enhanced the migration (Figure 2C, right) and the real-time cell migration rates of both HT29 and HCT116 cells (Figure 2D). Treatment with purified His-tagged syndecan-2 extracellular domain showed significant effects, on migration and anchorage-independent growth of colon cancer cells, without affecting cell proliferation (Supplementary Figure S3). Consistently, transfection with an Fc receptor-shed syndecan-2 chimera (sS2E-Fc) enhanced cell migration of HCT116 cells, sS2E-Fc proteins were detected in the conditioned media, and sS2E-Fc treatment enhanced cell migration and colony forming activities of HCT116 cells (Supplementary Figure S4). These data suggest that shed syndecan-2 extracellular domain contributes to syndecan-2-associated cancer activity regulation.

### Shed syndecan-2 synthetic peptide is sufficient for potentiating primary tumor growth and metastasis

**Figure 3:**
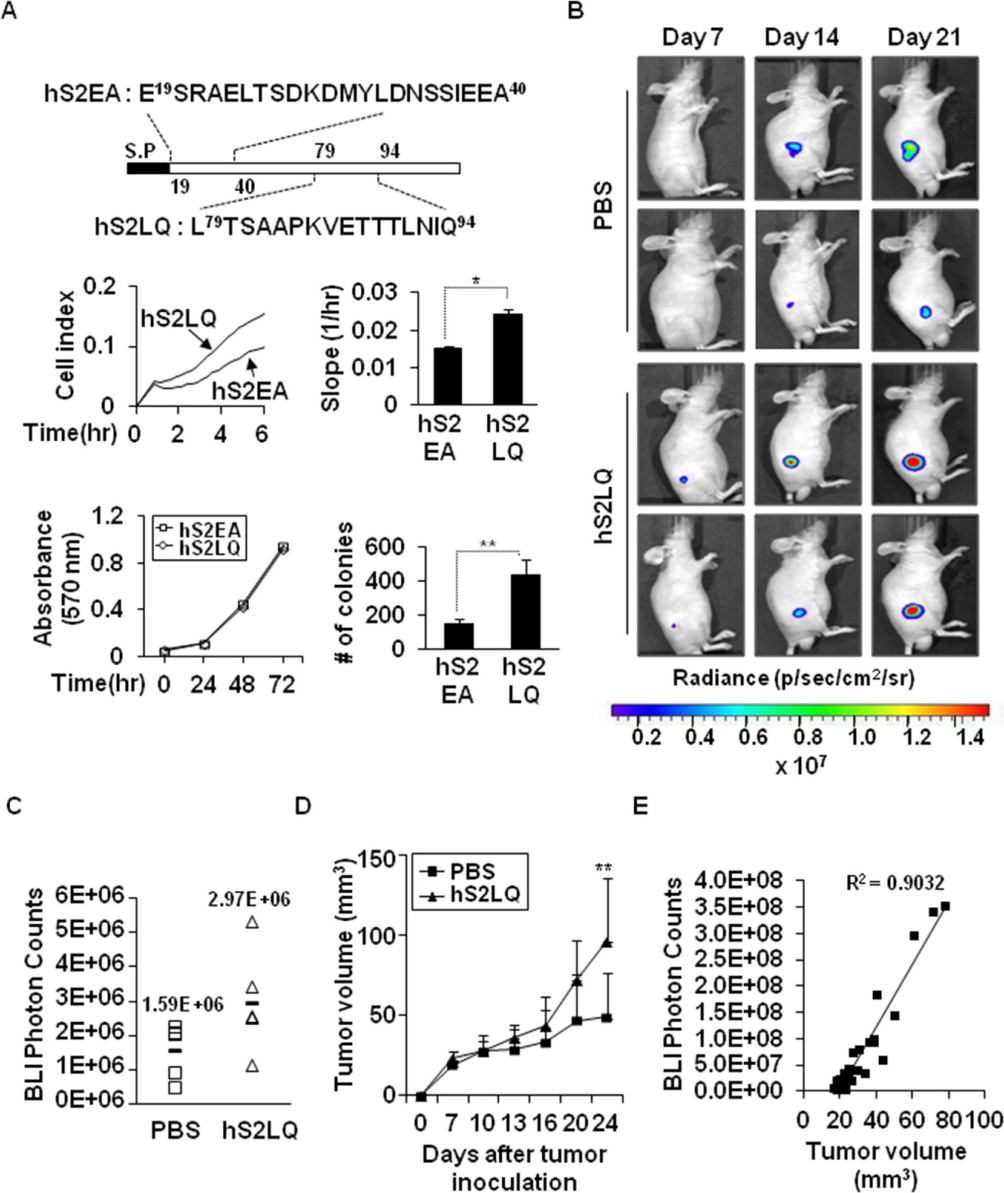
The syndecan-2 synthetic peptide significantly increases subcutaneous tumor growths *in vivo* **(A)** Schematic diagram depicting the synthetic peptides of human syndecan-2. Peptides corresponding to human syndecan-2 extracellular domain regions consisting of residues 79–94 (hS2LQ: LTSAAPKVETTTLNIQ) and residues 19–40 (hS2EA: ESRAELTSDKDMYLDNSSIEEA) were synthesized (top). HCT116 cells were mixed with synthetic peptides (final 5 nM). Cells migration was analyzed using the provided RTCA software (middle). *n* = 5; *p < 0.05. Cell viability was determined by MTT assay (bottom left). Colony formation was analyzed as described above (bottom right). N-terminal human syndecan-2 synthetic peptide (hS2EA) was used as a control peptide. *n* = 4; **p < 0.01. **(B)** HT29-luc cells (5 × 10^6^ cells/mouse) preincubated with synthetic peptides were injected subcutaneously into the right flanks of BALB/c nude mice (*n* = 5/group). Representative images of *in vivo* tumor development at the injection sites, which received HT29-luc cells incubated with human syndecan-2 synthetic peptide or PBS from the same mouse and taken weekly after implantation, are shown. **(C)** Tumor growth was quantified (as photon/s) weekly by IVIS at 7 days after injection. PBS, mean = 1.59E+06 photon/s, 95% CI = 1.35E+06 to 1.83E+06 photon/s; hS2LQ, mean = 2.97E+06 photon/s, 95% CI = 2.50E+06 to 3.44E+06 photon/s; *p* < 0.05. **(D)** Tumor volume (mm^3^) represented as average at 3–4 days. Average tumor volumes for 21 days were 42.75 mm^3^ with syndecan-2 peptide, 95% CI = 34.52 mm^3^ to 51.00 mm^3^ than the 29.21 mm^3^ with PBS, 95% CI = 24.94 mm^3^ to 33.48 mm^3^. **p < 0.01. **(E)** Correlation between tumor volume measurement and photon imaging (R^2^ = 0.9032).

We next constructed a series of recombinant deletion mutants of shed syndecan-2, a C-terminal deletion mutant, N2E-Fc, and an N-terminal deletion mutant, C2E-Fc of shed syndecan-2, expressed each in HEK293T cells, and collected the conditioned media. Treatment of HCT116 cells with the conditioned media containing C2E-Fc caused a remarkable increase in migration and anchorage-independent growth of HCT116 cells (Supplementary Figure S5A–S5C). When we further constructed a C2E-Fc deletion mutant, N-terminus residues 89–104 (L^89^TSAAPEVETMTLKTQ^104^, C2EQ^104^-Fc), the conditioned media from the C2EQ^104^-Fc-expressing cells enhanced migration of HCT116 cells (Supplementary Figure S5D), suggesting that the tumorigenic activity of shed syndecan-2 resides in the C-terminus of the extracellular domain. Expectedly, treatment of HCT116 cells with synthetic peptides corresponding to human sequence (hS2LQ) caused a remarkable increase in cell migration compared with the control peptide (hS2EA), without affecting cell proliferation (Figure 3A). In addition, hS2LQ-treated HCT116 cells showed a sharper increase in colony number following growth in soft agarose, compared to hS2EA-treated cells (Figure 3A). To further investigate the effect of syndecan-2 peptide on colon cancer cell primary growth, luciferase-expressing human colon cancer cells were subcutaneously injected into 6-week-old male BALB/c nude mice, and tumors were monitored and measured starting 7 days post injection for up to 21 days. Subcutaneous images showed an increase in photon emission from the tumor sites in syndecan-2 peptide treated mice compared with PBS treated control mice (Figure 3B and 3C). Moreover, mice given a syndecan-2 peptide developed more rapidly growing tumors than the PBS-treated mice (Figure 3D). There was a positive correlation between tumor weights and photon count (Figure 3E, R^2^ = 0.9032).

**Figure 4:**
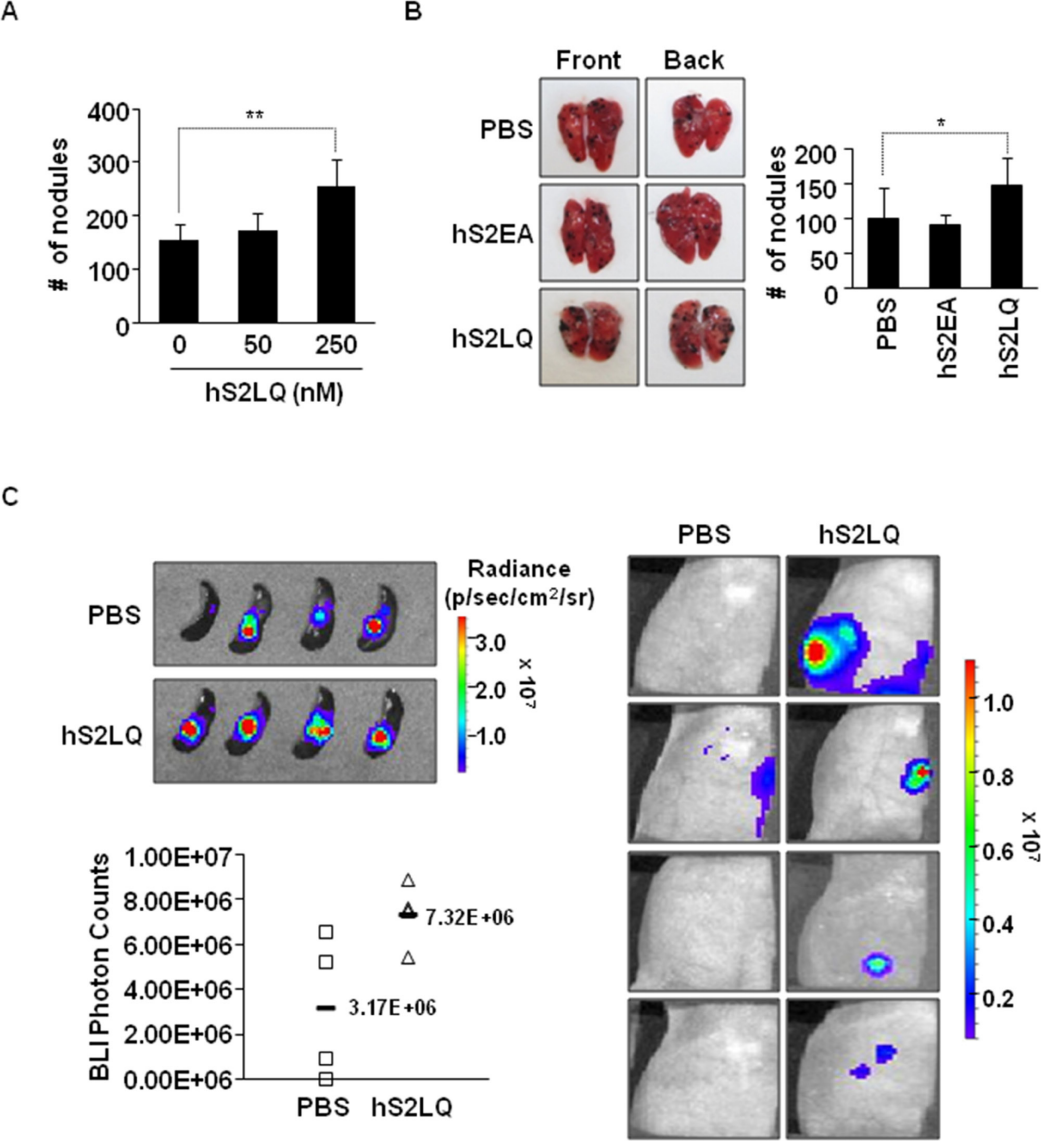
The syndecan-2 synthetic peptide significantly increases the metastasis in mouse models **(A)** BALB/c mice (*n* = 5/group) were injected with B16F10 melanoma cells (1 × 10^5^ cells/mice) incubated with the synthetic peptides into the tail vein. Mice were sacrificed after 2 weeks, and the number of metastatic tumor nodules was counted in the lungs. The bar graph indicates the numbers of metastatic lung nodules. The columns represent the mean ± s.d. of number of lung metastatic nodules, **p < 0.01. **(B)** B16F10 melanoma cells incubated with the indicated peptide (final 250 nM) were injected into BALB/c mice (*n* = 7/group) via the tail vein. Representative photographs of the front and back sides of each lung are shown. The columns represent the mean ± s.d. of number of lung metastatic nodules, *p < 0.05. **(C)** Mouse mammary cancer 4T1-luc cells (1 × 10^5^ cells/mice) were incubated with syndecan-2 synthetic peptide (final 250 nM) or PBS, and injected into spleens of BALB/c mice. Bioluminescence images of spleen (left, top; PBS, mean = 3.17E+06 photon/s, 95% CI = 2.10E+06 to 4.24E+06 photon/s; syndecan-2 peptide, mean = 7.32E+06 photon/s, 95% CI = 2.57E+06 to 12.07E+06 photon/s, *p* < 0.05) and liver (right) metastatic 4T1-luc cells, taken after 7 days of splenic injection, are shown. The respective photon counts of each mouse are represented by the color scales beside the mouse pictures. The IVIS imaging system acquired pictures were taken 10–20 min after intraperitoneal injection of d-luciferin (150 mg/kg). Quantification of tumor signal is represented as photon counts (left, bottom).

We further investigated the effects of the syndecan-2 peptide in a metastasis model. Firstly, B16F10 cells pre-incubated with different amounts of synthetic peptides of human syndecan-2 were injected into mouse tail veins and pulmonary metastasis was evaluated. Analysis of lung samples revealed that the synthetic peptide significantly and dose-dependently (Figure 4A) enhanced the pulmonary metastasis of B16F10 cells (Figure 4A and 4B). Secondly, we injected 4T1 cells stably transfected with luciferase into the spleen using a well-established mouse liver metastasis model and observed primary tumor growth and liver metastasis after 7 days. Syndecan-2 peptide significantly enhanced primary intrasplenic tumor growth, and liver metastasis of 4T1 cells (Figure 4C). Together, these *in vivo* results suggest that shed syndecan-2 directly plays an important role in primary tumor growth and metastasis.

### Elevated levels of shed syndecan-2 correlate with increased tumorigenic activity in the serum of patients with colon cancer

**Figure 5:**
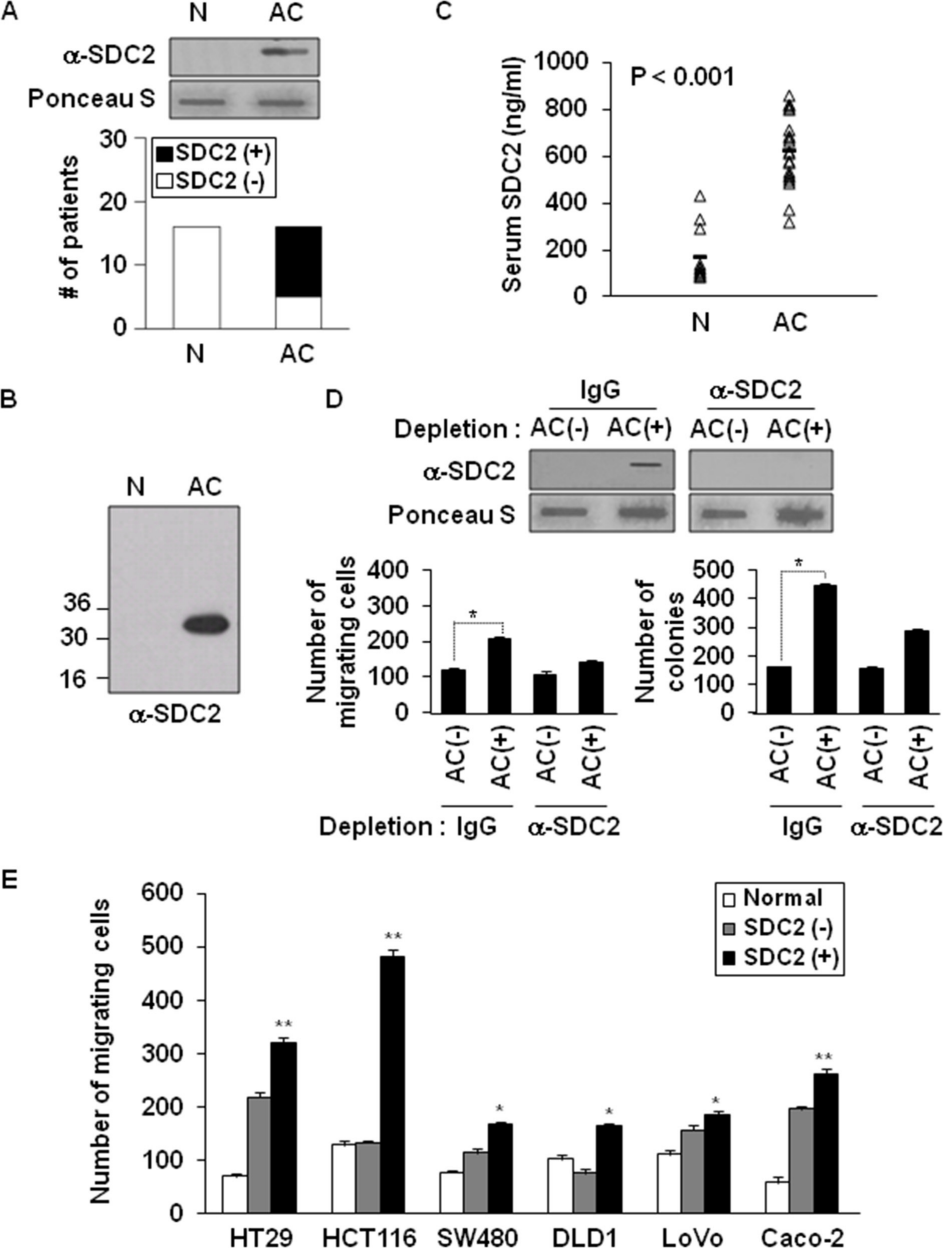
Elevated levels of shed syndecan-2 in serum correlate with increased migratory potential in colon cancer **(A)** Serum samples from either a normal donor (N) or advanced stages of colon cancer (AC) patients were subjected to slot blotting with anti-syndecan-2 antibody. The data represent the number of patients found to be serum negative (□) or serum positive (■) for the presence of shed syndecan-2. **(B)** Serum samples pretreated with HNO_2_ were resolved by 15% SDS-PAGE followed by blotting with the anti-syndecan-2 antibody. **(C)** Serum from patients with colon cancer (*n* = 12) and normal controls (*n* = 11) was analyzed by ELISA for shed syndecan-2 levels. Median levels are indicated by the horizontal bars. **(D)** Serum samples from shed syndecan-2 negative (AC−) and positive (AC+) AC patients were incubated with protein A Sepharose, and then the unbound materials were further immunodepleted with either IgG- or anti-syndecan-2 antibody-conjugated protein G Sepharose. The supernatants were subjected to slot blotting with the anti-syndecan-2 antibody (top). The supernatants were then used for Transwell migration assays and anchorage-independent growth assays with HCT116 cells (bottom). *n* = 4; *p < 0.05. **(E)** Colon cancer cells were treated with the indicated serum samples and allowed to migrate on gelatin-coated Transwell plates. *n* = 4; *p < 0.05, **p < 0.01.

We subsequently investigated whether shed syndecan-2 accumulated in serum from patients with colon cancer. Shed syndecan-2 was detected in 69% of advanced colon cancer patients (AC), but not in normal serum (Figure 5A). Similar results were obtained by western blotting (Figure 5B). ELISA assay with sera from the colon cancer patients revealed that levels of shed syndecan-2 in the sera were 625.9 ng/ml (range 321.2–863.6 ng/ml) in advanced colon cancer patients, whereas those of the sera from healthy people (N) was 176.3 ng/ml (range 87.4–431.0 ng/ml; Figure 5C). We then determined if shed syndecan-2 in sera from colon cancer patients could be related with colon cancer activity. Expectedly, shed syndecan-2-containing serum samples from patients were found to significantly enhance migration of HCT116 colon cancer cells compared to cells treated with serum samples with lower levels of shed syndecan-2 and depletion of shed syndecan-2 from the serum abolished the increased migration and anchorage-independent growth of colon cancer cells (Figure 5D). Interestingly, shed syndecan-2-containing sera from AC patients enhanced the migration of most of the tested colon cancer cell lines (Figure 5E). Together, these data suggest that shed syndecan-2-containing serum enhances tumorigenic activities in colon cancer cells.

### Shed syndecan-2 enhances MMP-7 expression via p38 MAP kinase activation in colon cancer cells

**Figure 6:**
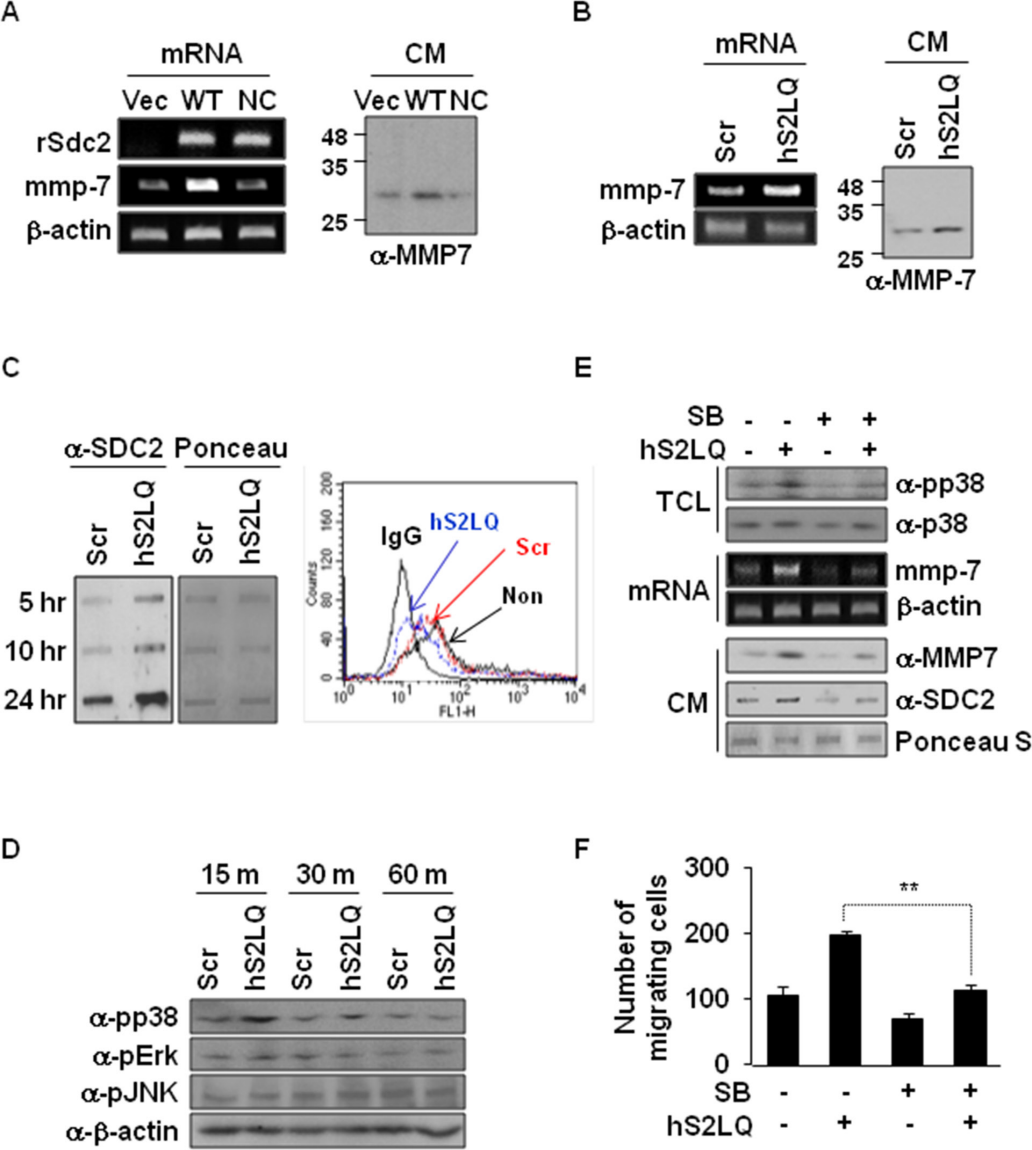
Shed syndecan-2 enhances MMP-7 expression via p38 MAP kinase activation in colon cancer cells **(A)** HT29 cells were transfected with indicated cDNAs. Expression of SDC2 and MMP-7 was analyzed by RT-PCR (left). Conditioned media (CM) were collected, and proteins were concentrated by TCA precipitation and analyzed by immunoblotting using anti-MMP-7 antibody (right). **(B)** HT29 cells were treated with either scrambled (Scr) or human (hS2LQ) peptide in SFM for 24 hr, and MMP7 expressions were analyzed by RT-PCR (left). Conditioned media were collected, and proteins were concentrated by TCA precipitation and analyzed by immunoblotting using anti-MMP-7 antibody (right). **(C)** HT29 cells stably overexpressing syndecan-2 were treated with the indicated peptides and conditioned media were subjected to slot blotting with the anti-syndecan-2 antibody (left). Cells were either untreated (non) or treated with the indicated peptides and cell surface expression of syndecan-2 was analyzed by Flow cytometry (right). **(D)** HT29 cells treated with the indicated peptides were lysed, and MAPK activation was assessed using an antibody specific for phospho-p38 (pp38). **(E)** HT29 cells preincubated with SB239063 (1 hr) were treated with the syndecan-2 synthetic peptide. After 15 min, total cell lysates (TCL) were subjected to immunoblotting with the indicated antibodies to determine the phosphorylation of p38 (top). At 6 hr, MMP-7 mRNA expression was analyzed by RT-PCR (middle), and conditioned media were subjected to either immunoblotting with anti-MMP-7 antibody or slot blotting with anti-syndecan-2 antibody (bottom). **(F)** HT29 cells preincubated with SB239063 (1 hr) were allowed to migrate on Transwell apparatus in the absence or presence of the syndecans-2 peptide. *n* = 4; **p < 0.01.

We finally investigated how shed syndecan-2 regulates tumorigenic activity in colon cancer cells. Consistent with the previous report [7], syndecan-2 overexpression in HT29 cells increased expression of MMP-7, an important regulator in syndecan-2-mediated tumorigenic activity, at the mRNA and protein levels (Figure 6A). Interestingly, however, this syndecan-2-mediated MMP-7 expression was dramatically reduced in cells expressing NC-SDC2 (Figure 6A), suggesting that shed syndecan-2 rather than syndecan-2 leads to increased expression of MMP-7. Indeed, the synthetic peptide (hS2LQ) caused a remarkable increase in mRNA and protein expression of MMP-7 in HT29 cells (Figure 6B). Consistently, treatment of HT29 cells with hS2LQ reduced cell surface expression of syndecan-2 and increased levels of shed syndecan-2 in the conditioned media (Figure 6C).

The mitogen-activated protein kinase (MAPK) signaling pathway is known to be involved in regulation of MMP-7 expression [26]. Compared with control cells, the synthetic peptide treatment significantly increased phosphorylation of p38 MAPK (Figure 6D). Consistently, when HT29 cells were pretreated with SB239063 (a specific inhibitor of p38 MAPK), we observed decreases in the synthetic peptide-mediated MMP-7 expression in parallel with decreased phosphorylation of p38 MAPK (Figure 6E) and decreased migration of HT29 cells (Figure 6F). These findings indicate that shed syndecan-2 regulates tumorigenic activity of colon cancer cells via p38 MAPK-mediated MMP-7 expression regulation.

## DISCUSSION

We previously reported that elevated expression of syndecan-2 potentiates the tumorigenic activity of colon carcinoma cells [7, 11, 27–29] but the exact molecular regulatory mechanism underlying this effect was not known. Since the functions of syndecan-2 are closely related to cell migration, it could be expected that syndecan-2 might play critical roles as an adhesion receptor. Indeed, syndecan-2 was found to modify integrin signaling, leading to enhanced cell adhesion and reduced cell migration [30, 31]. Notably, the present data show that syndecan-2 shedding is involved in the regulation of colon cancer cell migration. Increased cell migration was seen with wild-type (MMP-7-cleavable) syndecan-2, whereas a protease-resistant mutant triggered far less migration in human colon cancer cell lines and an animal model (Figure 1). Colon cancer cell migration was increased in response to treatment with exogenous shed syndecan-2 (Figure 2). These findings suggest that syndecan-2 shedding plays a role in regulating colon cancer cell migration. Previous reports have shown that the syndecan-2 extracellular domain as a substrate enhances attachment and focal adhesion formation in fibroblasts dependently on β1 integrin [31]. The syndecan-2 extracellular domain was shown to function as a novel ligand for CD148, in an interaction that stimulates β1 integrin-associated signal transduction and regulates cellular adhesion-related processes, such as angiogenesis and inflammation [31]. Together, these previous data support our contention that shed syndecan-2 plays a critical role in colon cancer cells. Interestingly, we found that the tumorigenic activity of shed syndecan-2 is associated with 16 amino acid residues in the C-terminus of the extracellular domain. As this is not the interaction site for CD148, our results suggest that unique regulatory mechanisms may be involved in syndecan-2 shedding and the associated regulation of cancer activity.

Our present work showed that the synthetic peptide induced MMP-7 upregulation and MMP-7-mediated syndecan-2 shedding (Figure 6), suggesting that syndecan-2 shedding may create a positive regulatory loop between syndecan-2 and MMP-7. Furthermore, the syndecan fragment generated by direct proteolytic cleavage could function as a cancer-specific ligand, triggering autocrine signaling through a yet-unidentified receptor. Indeed, shed syndecan-2 enhanced the tumorigenic characteristics of colon cancer cells in an autocrine manner (Figure 2), potentially affecting both the primary tumor growth (Figure 3) and the metastatic ability of colon cancer cells (Figure 4). Shed syndecan-2 may not directly regulate cell proliferation, since neither recombinant nor synthetic peptides were found to affect cell proliferation (Figure 3A and Supplementary Figure S3D). Since shed syndecan-2 was found to enhance the anchorage-independent growth of colon cancer cells (Figure 3A and Supplementary Figure S3E), we believe that the role of shed sydecan-2 is not limited to autocrine signaling. Treatment with exogenously shed syndecan-2 from HT29-cell-conditioned media enhanced the migration of HCT116 cells (Figure 2D), suggesting that shed syndecan-2 may also regulate the tumorigenic characteristics of colon cancer cells in a paracrine manner. In the same context, shed syndecan-2 may affect different cell types differently *in vivo*. Shed syndecan-2 located in the serum may circulate, allowing it to bind certain cells that have the appropriate receptor. A recombinant extracellular domain protein containing the sequence of shed syndecan-2 was previously shown to promote angiogenic processes in brain microvascular endothelial cells [21]. Therefore, shed syndecan-2 may enhance angiogenic processes, thereby facilitating cancer cells growth and metastasis.

Syndecan-2 shedding plays a key role in promoting tumorigenic activities of colon cancer cells. However, it remains unclear how shed syndecan-2 could regulate cancer associated activities. Moreover, studies to find specific strategies to disrupt the functions of shed syndecan-2 as a potential therapeutic approach for colon cancer also need to be continued. We suggest that anti-syndecan-2 antibody may provide a new therapeutic approach for diagnosis and treatment of colon cancer.

## MATERIALS AND METHODS

### Materials and antibodies

Monoclonal antibody against syndecan-2 that recognize human, rat, and mouse was produced by AdipoGen Inc. (Incheon, Korea) using Fc-fused syndecan-2 extracellular domain [25] and polyclonal anti-syndecan-2 antibody that recognize human, rat, and mouse was produced by AbClon (Seoul, Korea) using human syndecan-2 extracellular domain peptide. Monoclonal antibody against human MMP-7 purchased from Abcam (Cambridge, England), polyclonal antibodies to phospho-p38, phospho-JNK and p38 from Cell Signaling (Danvers, MA, USA), and monoclonal antibodies to phospho-Erk, and β-actin from Santa Cruz Biotechnology (California, CA, USA). Pro-MMP-7 enzymes were from Calbiochem (CA, USA), and Heparinase III/chondroitinase ABC and anti-FLAG M2 antibody were purchased from Sigma (St Louis, MO, USA).

### Cell lines and transfection

Cancer cell lines were purchased from the American Type Culture Collection (ATCC). HT29 and HCT116 cells were maintained in McCoy's 5A (Welgene, Daegu, Korea); DLD1, HEK293T, Caco-2 and B16F10 cells were maintained in DMEM (Welgene); LOVO cells were maintained in RPMI-1640 (Gibco BRL, NY, USA); and SW480 cells were maintained in DMEM-F12 (Welgene) complete media. The 4T1-luc and HT29-luc stable cells were grown in RPMI-1640 and McCoy's 5A media with puromycin (2 μg/ml). All media were supplemented with 10% fetal bovine serum (FBS) and gentamicin (50 μg/ml, Sigma), and grown at 37°C in 5% CO_2_ in a humidified atmosphere. Transient transfections were carried out using either the Lipofectamine 2000 (Invitrogen) reagent or the Effectene reagent (Qiagen, Hilden, Germany), according to the manufacturer's protocols.

### RT-PCR analysis

Total RNA extracted from cultured cells was used as template for reverse transcriptase reaction as described previously [23] using specific primers listed in Supplementary information.

### Migration and colony forming assays

Migration and colony forming assay were performed according to the standard protocol as described in Supplementary Information.

### Purification of soluble syndecans-2

HT29 cells were incubated in serum-free medium for 24 hr. The culture medium was harvested and centrifuged, and the supernatant was applied to a DEAE-sepharose column equilibrated with buffer 1 (50 mM Tris-HCl, pH 8.0, 4 M urea, 200 mM NaCl, 0.1% Tween-20, 10 mM NEM, 25 mM EDTA, and 2 mM PMSF). The column was washed with 10 column volumes of buffer 1 and then with 10 column volumes of buffer 2 (30 mM sodium acetate, pH 4.0, 4 M urea, 200 mM NaCl, 0.1% Tween-20, 10 mM NEM, 25 mM EDTA, and 2 mM PMSF, pH 4.0). Finally, the bound proteins were eluted with buffer 3 (150 mM sodium acetate, pH 4.0, 4 M GuHCl, and 0.1% Tween-20). Before resolved on SDS-PAGE, purified soluble syndecan-2 extracellular domain were incubated with a mixture of 2 mU heparinase III and 20 mU chondroitinase ABC for 8 hr at 37°C in heparinase reaction buffer (50 mM HEPES pH 6.5, 50 mM NaOAc, 150 mM NaCl, and 5 mM CaCl_2_).

### Peptide synthesis

Peptides corresponding to human syndecan-2 extracellular domain regions consisting of residues 79–94 (hS2LQ: LTSAAPKVETTTLNIQ) and residues 19–40 (hS2EA: ESRAELTSDKDMYLDNSSIEEA), and scrambled peptides for hS2LQ (Scr: IPNTSVKTLTAQLAET) were synthesized using an improved version of the solid-phase method utilizing Fmoc chemistry (Anygen Inc., Kwangju, Korea).

### Animals and human subjects

All animal experiments using BALB/c mice were conducted according to guidelines of Korean Institute of Science and Technology (KIST) under specific pathogen-free conditions in the KIST animal care facilities. Human sera of patients with from advanced carcinoma stages (*n* = 23), including from healthy donors (*n* = 23), were obtained from the Ewha Womans University School of Medicine from 2009 to 2011. Informed consent was obtained from all patients.

### Mouse tumor metastasis model

For the *in vivo* experimental pulmonary metastasis assay, both B16F10 melanoma cells (1 × 10^5^ cells/mouse) transfected with either wild type or non-cleaved mutant syndecan-2 and B16F10 cells incubated with human syndecan-2 peptide (final 50–250 nM) at 37°C for 30 min in 200 μl of PBS were injected via the tail veins into 6-week-old male BALB/c mice (*n* = 5–7 per group, Orient Bio Co., Seoul, Korea). On day 14, lungs were excised and metastatic nodules were photographed and counted. For the liver metastasis model, intrasplenic injection of mouse mammary tumor 4T1-luc stable cells (1 × 10^5^ cells/mouse) was done in 6-week-old female BALB/c mice (*n* = 8) along with human syndecan-2 peptide (250 nM). On day 7, primary tumor growth and development of liver metastasis were detected by the IVIS Imaging System (IVIS SPECTRUM, Caliper Life Sciences).

### Mouse subcutaneous tumor growth model

For the subcutaneous model, 6-week-old male BALB/c nude mice were given 150 mg/kg D-luciferin by intraperitoneal injection, and then anesthetized with 1% isoflurane. HT29-luc stable cells (5 × 10^6^ cells/mouse) incubated with human syndecan-2 peptide at 37°C for 30 min in 100 μl of PBS were subcutaneously injected below the dorsal flank of anesthetized animals. At 10–20 min after D-luciferin injection, mice were placed in the IVIS Imaging System and imaged dorsally. The images were captured on days 7, 14, and 21. Tumor growth was monitored weekly by IVIS and external caliper measurements were taken (L × W × D) for 24 days.

### Sandwich ELISA for shed syndecan-2

Costar® 96-Well EIA/RIA Stripwell™ Plate were coated with anti-syndecan-2 monoclonal antibody (2.5 μg/ml) in carbonate buffer (pH 7.4) as capture antibody and incubated at 4°C overnight. The wells were blocked for 2 hr with 5% non-fat milk in PBS at room temperature and human sera from colon cancer patients and healthy donors (0.1 μl/well) were placed in each antibody-coated well for 1.5 hr at 37°C. After washing, polyclonal anti-syndecan-2 antibody was diluted to the final concentration of 0.25 μg/ml in PBS, added to each well and incubated for 2 hr at room temperature. After washing, Goat-anti-rabbit IgG-HRP (1:10,000 dilutions) in blocking buffer was added and incubated for 1.5 hr. After a final washing, TMB substrate solution was added to each well and incubated for 25 min. The reaction was stopped by adding 0.5 M H_2_SO_4_ and the optical density were measured at 450 nm using a microplate spectrophotometer.

### Statistical analysis

All data are presented as mean ± s.d. Differences between groups were tested for statistical significance using Student's *t*-test and were considered significant at *p* < 0.05 or *p* < 0.01.

## SUPPLEMENTARY INFORMATION


