# Supplementary material for: Shed syndecan-2 enhances tumorigenic activities of colon cancer cells
Source: Oncotarget. 2015 Feb 17;6(6):3874–86. doi: 10.18632/oncotarget.2885 (PMC4414160; doi:10.18632/oncotarget.2885)
Supplement: Supplementary file 1 [file oncotarget-06-3874-s001.pdf]

SUPPLEMENTARY INFORMATION

Plasmid constructs

The vector construct encoding of Flag-tagged syndecan-2 was kindly provided by Dr. Yamaguchi Y. (Sanford-Burnham Medical Research Institute). In the current study, site-directed mutagenesis of the syndecan-2 extracellular proteolytic cleavage site [9] was performed using the QuikChange kit (Stratagene, CA, USA) according to the manufacturer's protocols. The Leucine residue at position 149 of the syndecan-2 extracellular domain was replaced with Isoleucine using the following primers that were deigned against the *syndecan-2* mRNA of Rattus norvegicus (GenBank accession number NM_013082.3): forward 5′- ACGTG TACACCGAGAAACATTCAGACAATATCTTCAAGC GG-3′ and reverse 5′-CCGTCCGCTTGAACGAATTGT CTGAATGTTTCTCGGTGTAC-3′.

Flow cytometry

Colon cancer cells were treated with indicated synthetic peptide for 24 hr, then washed with PBS and released 1 mM EDTA-5% FBS in PBS. After centrifugation, cells were resuspended in PBS and washed with PBS three times. Cells were incubated with anti-syndecan-2 monoclonal antibody in 10% FBS in PBS for overnight at 4°C. Followed by washing three times with PBS containing 0.05% Tween-20, cells were incubated FITC-conjugated anti-mouse antibody in 10% FBS in PBS for 1 hr at 25°C. Syndecan-2 expression on cell surface was analyzed by flow cytometry.

RNA extraction and Reverse Transcription Polymerase Chain Reaction (RT-PCR)

Total RNA extracted from indicated cells was used as template for reverse transcriptase reaction. Aliquots of cDNA were amplified using the following primers: Rat syndecan-2 (forward) 5'-ATGCGGGTACGAGCCACGTC-3' and (backward) 5'- CGGGAGCAGCACTAGTGAGG-3', mouse syndecan-2 (forward) 5′-TGGCTACTTCGTTT GAGCAC-3′ and (backward) 5′-CACTACATTCTCAG CCTCCG-3′; human MMP-7 (forward) 5'-GGTCACCT ACAGGATCGTATCATAT-3' and (backward) 5'-CA TCACTGCATTAGGATCAGAGGAA-3', human β-actin (forward) 5'-TGGAATCCTGTGGCATCCATGAAA-3' and (backward) 5'-TAAAACGCAGCTCAGTAACAG TCCG-3'. After an initial denaturation at 94°C for 5 min, 20-30 cycles of denaturation at 94°C for 30 sec, annealing at 55°C for syndecan-2, 58°C for human MMP-7 and 52°C for human β-actin for 30 sec, and extension at 72°C for 60 sec for syndecan-2 or 30 sec for human MMP-7 and human β-actin were carried out. The reaction products were analyzed in 1% agarose gels.

Slot blotting

Either conditioned media or serum of cancer patients was slot-blotted onto a nitrocellulose membrane in a Bio-Rad apparatus (Bio-Rad, Hercules, CA, USA) and washed once with PBS. The membrane was then blocked in 0.05% Tween-20 in Tris-buffered saline (TBS: 50 mM Tris-HCl, pH 7.4, 150 mM NaCl) with 5% skim milk, washed, and probed with appropriate primary antibodies for 24 hr at 4°C, followed by species-specific horseradish peroxidase-conjugated secondary antibodies from AbClon. The signals were detected by enhanced chemiluminescence (ECL; AbClon).

Migration assay

For Transwell migration assay, the lower surface of Transwell inserts (Corning) was coated with gelatin (10 µg/ml), and the membranes were allowed to dry for 1 hr at room temperature. The Transwell chambers were assembled on a 24-well plate, and the lower chambers were filled with fibroblast growth factor-2 (100 ng/ml) in fresh media. Cells were added to each upper chamber, and the plates were incubated at 37°C in 5% CO_2 for 16 hr (HCT116 cells), 30 hr (HT29 cells), and 6 hr (B16F10 cells). The cells that had migrated to the lower surface of the filters were stained with 0.6% hematoxylin and 0.5% eosin, and counted. Four visual fields were randomly chosen, and the migrated cells were counted under a light microscope. The data are presented as the average cell number per field. For the studies of cell migration in real time, the xCELLigence system (Roche Diagnostics GmbH, Switzerland) was used. To examine cell migration, the lower chambers of a CIM-plate 16 (8-µm pore size) were filled with fresh medium containing 10% FBS. The upper chambers were filled with serum-free medium (30 µl/well), and the plate was incubated at 37°C in 5% CO_2 for 1 hr. The background was measured using the RTCA DP Analyzer. The HCT116 (2.5×10^4 cells/well) or HT29 cells (3×10^4 cells/well) were added to each well, and the plate was incubated at 25°C. After 30 min, the CIM-plate was assembled onto the RTCA DP Analyzer, and cell migration was assessed at 5 min intervals at 37°C in 5% CO_2. The obtained data were analyzed using the provided RTCA software.

Purification of the recombinant 6-His-tagged syndecan-2 extracellular domain

The expression vector pET32a-syndecan-2 extracellular domain (His-SDC2E, amino acids 29-154) was established and transformed into E. coli BL21 (DE3). Cells were allowed to grow until they reached the optical density of around 0.6-0.8 at 600 nm and induced by adding isopropyl β-d-1-thiogalactopyranoside (IPTG) to a final concentration of 0.2 mM for 16 hr at 25°C. The expression of Tobacco Etch Virus (TEV) protease was induced by adding IPTG to a final concentration of 1 mM for 6 hr at 37°C. After induction with IPTG, the cells were harvested by centrifugation at 6,000 xg for 30 min and resuspended in lysis buffer (20 mM sodium phosphate buffer pH 7.5 containing 500 mM NaCl, 5 mM β-mercaptoehanol, 1% TX-100), and then lysed by sonication on ice. The lysates was centrifuged at 14,000 xg for 30 min and supernatant was loaded onto Ni-NTA affinity column (Qiagen) according to the protocol specified by the manufacturer. The loaded column was washed with lysis buffer with 30 mM imidazole and His-SDC2E was eluted with the elution buffer (20 mM sodium phosphate buffer pH 7.5 containing 300 mM NaCl, 5 mM β-mercaptoehanol, 500 mM imidazole, 1% TX-100, pH 7.5). The elution fraction was dialyzed and incubated with TEV protease for 6 hr at 25°C. The digested products were purified through Ni-NTA affinity column and analyzed by SDS-PAGE.

Cell proliferation assay

Proliferation of HCT116 cells was measured by a colorimetric assay using 3-(4,5-dimethylthiazol-2-yl)-2, 5-diphenyltetrazolium bromide (MTT) as described [29] in the presence or absence of purified syndecan-2 fragments. This assay was tested on three separate occasions with triplicate samples, and the absorbance values were averaged.

Anchorage-independent growth in soft agarose

Each well of a 6-well culture plate was coated with 3 ml of bottom agar mixture (McCoy's 5A, 10% FBS, 0.6% agar). After the bottom layer had solidified, 1 ml of top agar mixture (McCoy's 5A, 10% FBS, 0.3% agar) containing either HCT116 or HT29 cells (1×10^5 cells/well) was added to each well with various concentrations of shed syndecan-2 fragments, and the cultures were incubated at 37°C in a 5% CO_2 atmosphere. Colony formation was monitored daily with a light microscope. Colonies in soft agar were photographed after incubation for 14 days with a digital camera. Colonies were fixed with methanol, stained with 0.1% crystal violet, and colonies with a diameter greater than 20 μm were counted in each well under a microscope.

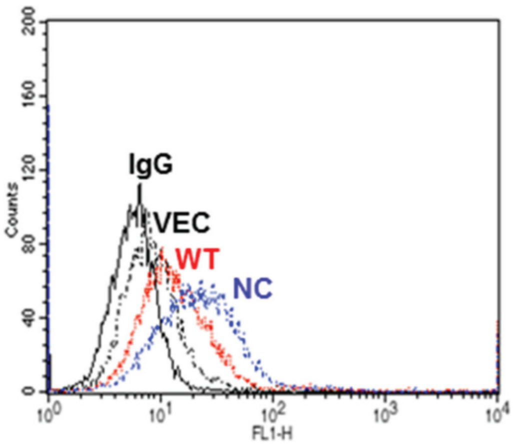

Supplementary Figure S1: Cell surface expression of syndecan-2 is higher in transfected cells expressing the non-cleavable mutant. HT29 colon cancer cells were transfected with 1 μg of VEC, WT- or NC-syndecan-2. Cell surface expression of syndecan-2 was analyzed by Flow cytometry.

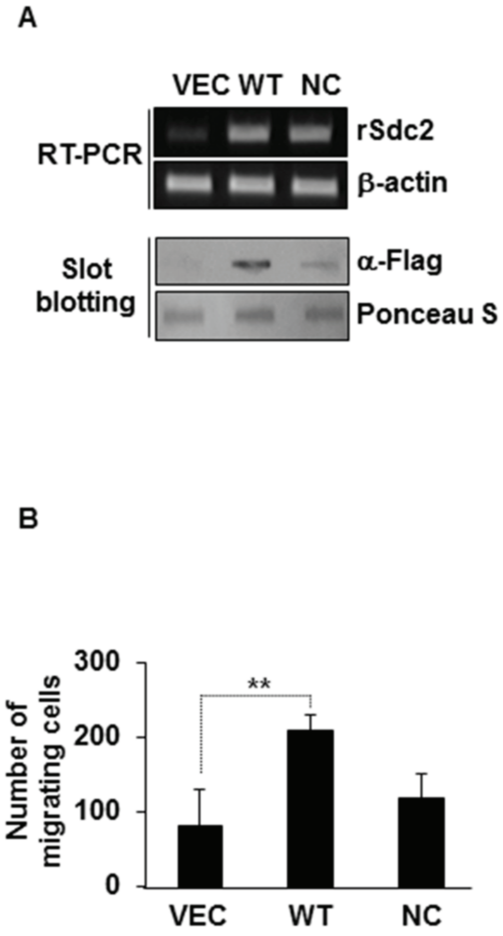

Supplementary Figure S2: Extracellular domain shedding of syndecan-2 affects melanoma cell migration. (A) B16F10 melanoma cells were transfected with VEC, Flag-WT- or -NC-syndecan-2. Syndecan-2 mRNA expression was evaluated by RT-PCR using the indicated primer using β-actin as a control (top). Conditioned medium was subjected to slot blotting with the anti-Flag antibody. Ponceau S staining was done to determine loading for protein blots (bottom). **(B)** Transfected cells were allowed to migrate on gelatin-coated (10 μg/ml) Transwell plates, and migrated cells were stained with hematoxylin and eosin. Data are shown as mean ± s.d., $n = 3$; $^{**}p < 0.01$.

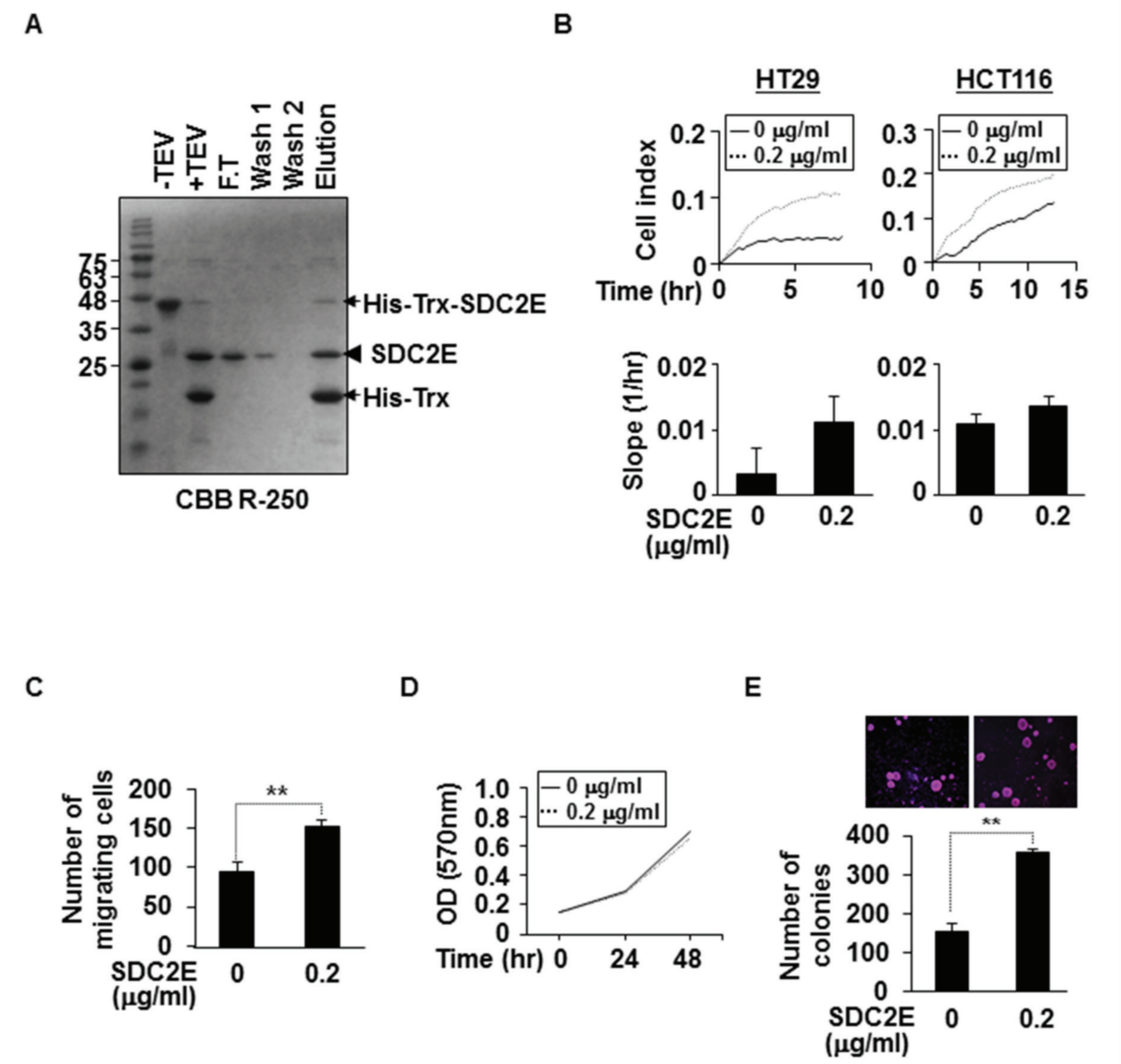

Supplementary Figure S3: Recombinant shed syndecan-2 enhances tumorigenic activities of colon cancer cells. **(A)** His-tagged thioredoxin (Trx)-syndecan-2 extracellular domain (His-Trx-SDC2E) fusion proteins were affinity purified over Ni-NTA columns and incubated with TEV protease. Each fractions and cleavage products were separated by 15% SDS-PAGE and CBB R-250 stained. Arrowhead indicates His-removed SDC2E, arrows indicate His-Trx-SDC2E or His-Trx. **(B)** HT29 and HCT116 cells were treated with 0.2 μg/ml of purified recombinant SDC2E, and allowed to migrate on RTCA CIM-Plate wells. **(C)** HCT116 cells were treated with 0.2 μg/ml of purified recombinant SDC2E, and allowed to migrate on Transwell apparatus. Data are shown as mean ± s.d., $n = 3$; $^{**}P < 0.01$. **(D)** HCT116 cells were treated with 0.2 μg/ml of purified recombinant SDC2E, and cell numbers were evaluated with MTT assay for the indicated times. The experiments were performed in duplicate, with triplicate samples. **(E)** Mixture of indicated cells (1×10^5 cells/well) with 0.2 μg/ml of purified recombinant SDC2E was added to bottom agar and colonies were counted after 2 weeks. Data are shown as mean ± s.d., $n = 3$; $^{**}P < 0.01$.

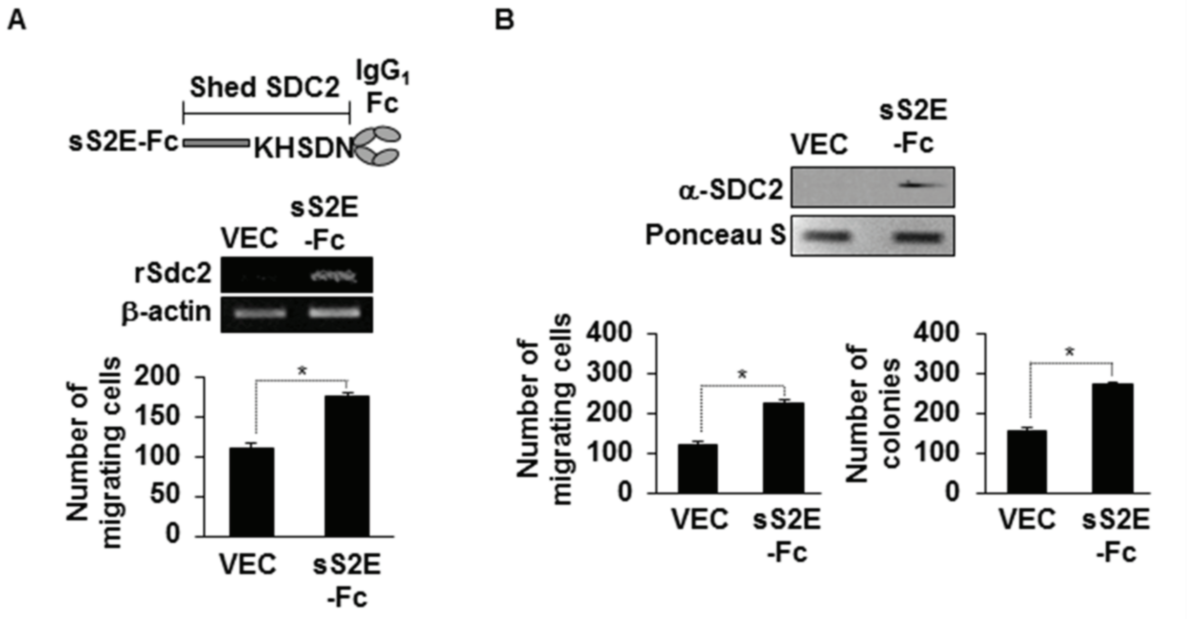

Supplementary Figure S4: Treatment with exogenous recombinant shed syndecan-2 stimulates tumorigenic activities of colon cancer cells. **(A)** HCT116 cells were transfected with either VEC or the Fc receptor-shed syndecan-2 chimera (sS2E-Fc, top), and syndecan-2 mRNA expression was evaluated by RT-PCR (middle). Transfected cells were allowed to migrate on Transwell plates (bottom). Data are shown as mean ± s.d., $n = 5$; *$p < 0.05$. **(B)** VEC- or sS2E-Fc-expressed conditioned media from HCT116 cells were subjected to slot blotting with anti-syndecan-2 antibody (top). HCT116 cells treated with the indicated conditioned media (final 10% v/v) were allowed to migrate on Transwell plates (bottom, left). HCT116 cells treated with the indicated conditioned media (final 10% v/v) were added to bottom agar, and the number of viable colonies was counted after 2 weeks (bottom, right). Data are shown as mean ± s.d., $n = 5$; *$p < 0.05$.

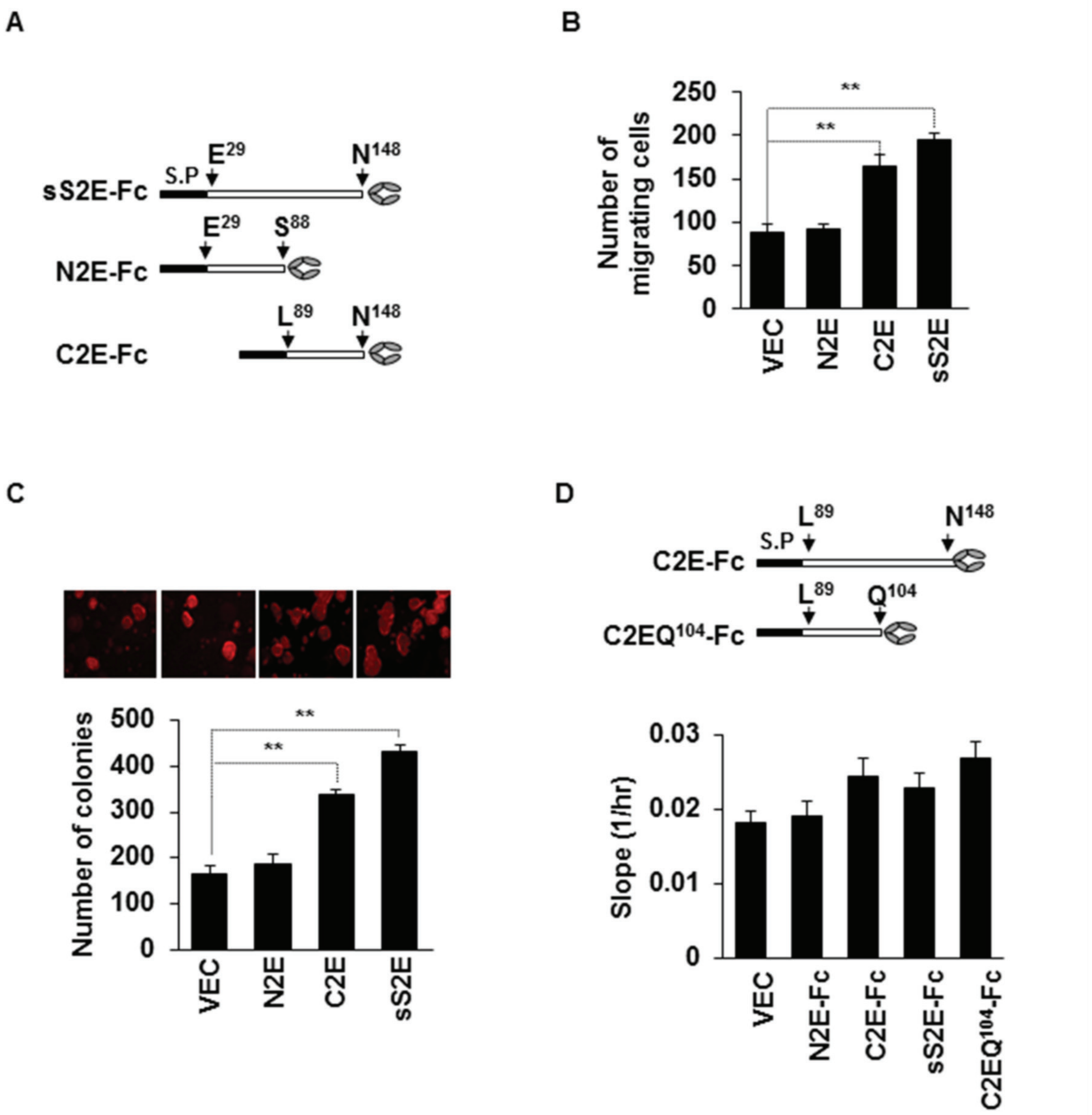

Supplementary Figure S5: The C-terminal region of the shed syndecan-2 enhances tumorigenic activities of colon cancer cells. **(A)** Schematic of structure of Fc receptor-shed syndecan-2 chimera (sS2E-Fc). N-terminal region (residues 29-88, N2E-Fc), C-terminal region (residues 89-148, C2E-Fc), and signal peptide (residues 1-28, S.P) are indicated. Syndecan-2 extracellular domain-Fc chimera was constructed to the Fc portion of human IgG_1. The syndecan-2 PCR products were inserted into Fc portion contained pcDNA 3.1 expression vector by digestion with EcoRI/XhoI enzyme site. **(B)** HEK293T cells were transfected with indicated cDNAs, and mixtures of HCT116 cells (1×10^5 cells/well) with the indicated HEK293T conditioned media (final 10% v/v) were allowed to migrate on Transwell plates. Data are shown as mean ± s.d., $n = 3$; $^{**}p < 0.01$. **(C)** HCT116 cells prepared described as B were added to bottom agar, and colonies were counted after 2 weeks. Clonogenicity was calculated as percent colonies formed in relation to the VEC control. Data are shown as mean ± s.d., $n = 3$; $^{**}p < 0.01$. **(D)** Schematic of structure of Fc receptor-C-terminal (residues 89-148, C2E-Fc) and C2EQ^{104}-Fc (residues 89-104) are indicated. HEK293T cells were transfected with indicated cDNAs, and mixtures of either HCT116 cells (2×10^4 cells/well), along with each conditioned media (final 10% v/v), and were added to each RTCA CIM-Plate well. The plate was incubated for 6 hr and analyzed using the provided RTCA software.
